# CERVIXNET: An Efficient Approach for the Detection and Classifications of the Cervigram Images Using Modified Deep Learning Architecture

**DOI:** 10.2174/0115734056343690250116020310

**Published:** 2025-01-23

**Authors:** N. Karthikeyan, Gokul Chandrasekaran, S. Sudha

**Affiliations:** 1 Department of Electronics and Communication Engineering, Velalar College of Engineering and Technology, Thindal, Erode 638012, India; 2 Department of Electronics and Communication Engineering, Karpagam Institute of Technology, Coimbatore 641105, India; 3 Department of Electronics and Communication Engineering, Kongu Engineering College, Perundurai, Erode 638060, India

**Keywords:** Cervical, Cancer, Deep learning, Cervigram, CervixNet, Architecture

## Abstract

**Introduction:**

The earlier detection of cervical cancer in women patients can save human life. This article proposes a novel methodology for detecting abnormal cervigram images from healthy cervigram images and segments the cancer regions in the abnormal cervigram images using the deep learning method. The conventional deep learning architecture has been modified into the proposed CervixNet architecture to improve the cervical cancer detection rate.

**Methods:**

This methodology is constituted of a training and testing process, where the training process generates the training sequences individually for healthy cervigram images and the cancer case cervigram images. The testing process tests the cervigram images into either a healthy or cancer cases using the training sequences generated through the training process. During the testing process of the proposed system, the cancer segmentation algorithm was applied on the abnormal cervigram image to detect and segment the pixels belonging to cancer. Finally, the performance has been carried out on the segmented cancer cervical images for the ground truth images. This proposed methodology has been evaluated on the cervigrams on IMODT and Guanacaste databases. Its performance has been analyzed concerning cancer pixel sensitivity, cancer pixel specificity and cancer pixel accuracy.

**Results:**

This research work obtains 98.69% Cancer Pixel Sensitivity (CPS), 98.76% Cancer Pixel Specificity (CPSP), and 99.27% Cancer Pixel Accuracy (CPA) for the set of cervigram images in the IMODT database. This research work obtains 99.22% CPS, 99.03% CPSP, and 99.01% CPA for the set of cervigram images in Guanacaste database.

**Conclusion:**

These experimental results of the proposed work have been significantly compared with the state-of-the-art methods and show the significance and novelty of the proposed works.

## 1 INTRODUCTION

The irregular pattern of the cells in the human body creates the cancer diseases. Due to this irregular pattern of the cells, the mitosis process is affected which creates more cancer cells rapidly in the human body. These cancer cells affect the nearby cells and spread to the other nearby organs which kills the human life based on their immunity levels [1-3]. Though the cancers affect all humans irrespective of their sexual gender type, breast and cervical cancers occur only in women patients around the world. Apart from the cancers that occurred on the men patients, the spreading ratio of cancers in women patients is relatively higher than the cancers spreading ratio in men patients. Hence, it is very important to identify the cancers occurred in women patients [4-7]. The cancers are formed in the breasts of the women patients and it can be easily detected by initial physical examination. The woman patient life can be saved by removing the cancer-affected breast portions. After the surgery, the survival rate of the women patient is extended. The cancer occurs in the cervix region of the women patient body is the main reason for forming cervical cancer. The formation of the Human Papilloma Virus (HPV) in the cervix region is the main causes of cervical cancer. There are four stages of cervical cancer that are identified in women patients as Stage I, Stage II, Stage III, and Stage IV. The first two stages of cervical cancer are identified as mild stage and they can be cured by taking proper medication. Stage III is identified as moderate and the spreading ratio of the cancer cells in this case is relatively high when compared with the previous two cervical cancer stages. The slight bleeding of blood from the cervix region has started at this stage and the women patient feels pain in the abdomen region. Stage IV is identified as the severe one and the spreading ratio of the cancer cells in this case is relatively very high when compared with the previous three cervical cancer stages [8-12]. The heavy bleeding of blood from the cervix region has started at this stage and the women patient feels the very pain at the abdomen region. Death can occur at this stage. Hence, the earlier detection of cervical cancer in women patients is very important than the detection of breast cancer. This motivates to bring this research work to detect cervical cancer in women patients.

Cervical cancer in women patients can be detected by both Pap smear cell test and Cervigram test [13-15]. In the case of the Pap smear cell test (as illustrated in Fig. (**1a**), the cells from the cervix region have been captured and their morphological characteristics have been analyzed. In the case of Cervigram test (as illustrated in Fig. (**1b**), the cervical images have been obtained from the cervix region and they have been synthesized by the various image processing methods [16]. In this article, a modified and enhanced deep learning methodology has been proposed to detect the cancer cervical images from the healthy cervical images.

The motivation of this paper is stated as follows.

The manual detection of cervical cancer through the expert physician or radiologist in women patients leads to death. In India such as high population countries, there are lack of experienced radiologists and physician to detect cervical cancer in a large population [17, 18]. This delays the cervical cancer detection and screening process. Moreover, the cervical cancer detection accuracy is not optimum due to the manual intervention [19-21]. These reasons motivate us to develop this paper using fully automated computer-based cervical cancer screening methods.

The problem definition of this paper is stated as follows.

- All the previous traditional cervical cancer detection methods are based on machine learning models which required more number of cervical images in both training and testing cases of the classification process. This sometimes causes overfitting issues [22, 23] during the classification process.
- The traditional classification algorithms have design complexity which consumes more classification time for differentiating the healthy cervical image from the cancer case cervical images [24].
- The cervical cancer detection accuracy is not optimum for severity determination [25].

These problems of traditional cervical cancer detection methods have been overcome by the methodologies stated in this paper.

The main contributions of this work are stated as follows.

- The noise contents will be detected and reduced in the cervigram images using Directional Noise Reduction Filter.
- The Gabor Multiresolution Transform is used for spatial-multi scale frequency conversion of all pixels in the noise-reduced cervical image.
- The multi-level features are determined from the Gabor Multiresolution transformed cervical image.
- These multi-level features are fed into the proposed CervixNet classifier for producing the classification results.
- The morphological algorithms have been used on the abnormal cervical image to locate the cancer pixels.

The novelty of this paper is given as,

- A unique Directional Noise Reduction Filter (DNRF) is proposed to detect and reduce the noise components in the cervigram images.
- The higher efficient and fast CervixNet classification architecture has been proposed which contains two parallel layering modules for obtaining the higher cervical image classification results.

This paper is organized into various sub-sections, as section 2 details the methodologies that belong to existing cervical cancer detection systems, section 3 proposes a novel CervixNet classification architecture for detecting cervical cancer and section 4 discusses the experimental results of the CervixNet on a different dataset. Section 5 depicts the conclusion of this article by stating the main outcomes of this research work.

## 2 LITERATURE SURVEY

Shtwai Alsubai *et al*. (2023) screened cervical cancer with the aid of Pap smear cell images [26]. In this work, the Pap smear cell images have been taken from the cervix region and their internal morphological properties were analyzed. Based on the morphological values of the obtained Pap smear cell images concerning clinical evaluation results, the Pap smear cell-based cancer regions have been identified in this work. The authors used linear regression-based CNN methodology for detecting and classifying the abnormal Pap smear cell images in this work. Moreover, the proposed works based on Pap smear cell images have been tested with a large cervical imaging dataset to evaluate their experimental and clinical results. The authors obtained 95.29% CPS, 96.39% CPSP and 95.30% CPA on IMODT database and also obtained 97.20% CPS, 96.39% CPSP and 96.28% CPA on Guanacaste database. Nitin Kumar Chauhan *et al*. (2023) concatenated the intrinsic and extrinsic multi-level and multi-class features for the detection and classifications of the cervical images [27]. The hybrid CNN architecture have been used in this work to differentiate the healthy cervical images from the cancer cervical images. Feature fusion was performed in this work to improve cervical cancer detection accuracy. The authors obtained 94.3% CPS, 94.29% CPSP and 94.94% CPA on the IMODT database and also obtained 93.28% CPS, 93.09% CPSP, and 93.27% CPA on Guanacaste database. Sehra *et al*. (2023) developed artificial intelligence-based cervical cancer detection methods using cervix images [28]. The different functional artificial intelligence methods were applied on the cervix images and the results of these methods were compared based on the morphological parameters. The authors obtained 94.27% CPS, 95.86% CPSP and 94.57% CPA on IMODT database and also obtained 96.75% CPS, 96.20% CPSP and 95.95% CPA on Guanacaste database.

Umesh Kumar Lilhore *et al*. (2022) integrated the causal analysis technique along with the machine learning methodology to detect cervical cancer images from healthy cervical images [29]. This hybrid technique changed the detection capability of the lower-resolution cervical images. The texture features along with the variable features have been derived from the cervical images and these were characterized by the causal analysis technique and then the evaluated results were classified further by the machine learning technique in this work. The authors obtained 93.97% CPS, 93.29% CPSP and 93.98%CPAon IMODT database and also obtained 94.28% CPS, 94.57% CPSP and 94.98%CPA on Guanacaste database.

Saini *et al*. (2022) applied transfer learning methodology for the detection and classification of cervical images [30]. The multi-class properties of the cervix regions have been derived and classified further through the non-linear transfer learning incorporated machine learning techniques. This proposed method has been evaluated on multi-class imbalanced large cervical imaging dataset and the experimental results were compared more significantly with their corresponding clinical evaluation results. The authors obtained 94.20% CPS, 94.47% CPSP and 93.29% CPA on IMODT database and also obtained 95.30% CPS, 95.95% CPSP and 94.39% CPA on Guanacaste database.

Zaid Alyafeai *et al*. (2020) designed a flexible and efficient cervical cancer detection methodology using pipeline architecture [31]. This complete automated cervical cancer detection method detected and segmented the abnormal region of pixels which were belonging to the cancer category using the integration of machine and deep learning methods. The authors obtained 95.29% CPS, 94.67% CPSP and 94.29% CPA on IMODT database and also obtained 94.29% CPS, 94.08% CPSP and 93.57% CPA on Guanacaste database. Elayaraja *et al*. (2018) implemented radial and feed-forward back propagation neural networks on the set of both healthy and cancer cervical images to differentiate them which were based on the morphological parameters [32]. This method used unsupervised neural networks in both categories of cervical differentiation and the experimental results of this method have been significantly compared with the clinical evaluation results. The authors obtained 93.2% CPS, 93.98% CPSP, and 93.27% CPA on IMODT database and also obtained 93.28% CPS, 93.14% CPSP and 93.29% CPA on Guanacaste database.

## 3 PROPOSED METHODOLOGIES

This article proposes a novel methodology for detecting abnormal cervigram images from healthy cervigram images and segments the cancer regions in the abnormal cervigram images. This methodology is constituted of a training and testing process, where the training process generates the training sequences individually for healthy case cervigram images and the cancer case cervigram images. The testing process tests the cervigram images into either a healthy case or a cancer case using the training sequences that are generated through the training process. Fig. (**2a**) is the cervigram image classification system in the case of the training process and Fig. (**2b**) is the cervigram image classification system in the case of the testing process. During the testing process of the proposed system, the cancer segmentation algorithm was applied to the abnormal cervigram image to detect and segment the pixels belonging to cancer. Finally, the performance has been carried out on the segmented cancer cervical images concerning the ground truth images.

### 3.1 Directional Noise Reduction Filter (DNRF)

The cervigram images may have certain noise content during the image acquisition process in scanners. These noise contents may create false identification of the cancer pixels in the cervigram images. To overcome this false identification issue in the cervigram images, the noise contents should be detected and reduced in the cervigram images before it is processed to the next stages. The existing filters such as Averaging filter, Median filter, and Adaptive median filters filtered the entire cervigram image without checking whether the pixels were being affected by noise or not. This will damage the normal pixels in the cervigram image which also affects the further cancer detection process. Hence, there is a requirement for developing an effective filter that performs the detection process initially and the filtering process have been applied if the noise is detected in the pixel of the cervigram image. This brings us to develop the DNRF to detect the affected noise pixel in the cervigram image. This proposed DNRF algorithm checks whether the pixel is being affected by the noise or not. If it is affected, then the filtering operation starts, else the pixel is not filtered by this DNRF.

This proposed DNRF has been explained in the following steps.

**Algorithm: DNRF**

**Input:** Cervigram with noise;

**Output:** Filtered cervigram image;

**Start;**

**Step 1:**

Place odd-numbered kernel window on the cervigram image. In this article, an 11*11 kernel window has been fixed over the cervigram image and the center pixel of this window has been chosen as the pixel to be checked for noise content.

**Step 2:**

Within the kernel window, select four direction-placed pixels such as horizontal, vertical, and two diagonal and they are depicted by the following equations.

**Table:** 

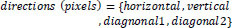	(1)

**Step 3:**

Perform ascending order of all pixels [33] in individual directions of pixels;

**Step 4:**

Remove the center pixel in all directions of pixels which is going to be checked for noise content;

**Step 5:**

Remove the first and last pixel values in the sorted direction of pixels;

**Step 6:**

Find the individual standard deviation value of the individual direction of pixels in step 5.

**Step 7:**

Choose the particular direction of the pixel with the lowest standard deviation value.

**Step 8:**

Find the Heuristic Metric (HM) [34] for the pixels in the particular direction of pixels using the following equation.

**Table:** 

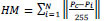	(2)

Whereas, *P_c_* is the center pixel in the particular direction of pixels, *P_i_* is the pixels in the particular direction of pixels and N is the number of pixels in the particular direction of pixels.

Step **9:**

The noise content of the pixel is identified based on the value of HM as depicted by the following equation.

**Table:** 

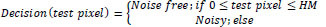	(3)

**Step 10:**

If the decision of the test pixel is noisy, then only apply an averaging filter [35] to remove the noise content in the pixel.

**End;**

### 3.2 Gabor Multiresolution Transform (GMT)

The noise content removed cervigram images have the pixel mode for time and amplitude. Due to this pixel mode behavior, certain features will not be extracted from these time sequenced pixels in the cervigram. Hence, the pixels in the cervigram image have to be changed into frequency series so that the features can be extracted from the frequency series cervigram image. Though conventional transforms such as Discrete Wavelet Transform (DWT), Radon, and Shearlet transforms [23] are available for this time series into frequency series pixel conversion process, the error rate during the pixel series transformation process is high. This creates a serious impact on the cervigram detection process. To overcome such limitation of error rates during the pixel transformation process, this proposed system uses GMT for the effective pixel transformation from time series to frequency series [26].

The GMT has functioned through the Gabor Filter (GT) with various frequency modes of operation and scales. The GT of the GMT is depicted in the following equation.

**Table:** 

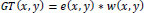	(4)

**Table:** 

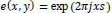	(5)

**Table:** 

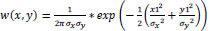	(6)

**Table:** 

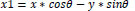	(7)

**Table:** 

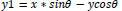	(8)

Whereas, *θ* is the pixel angle for x and y direction and s is the scale variable.

The pixel angle and scale variable determine the scalability of the transformation process. In this article, the pixel angle is kept from -90 degrees to +90 degrees by the angle increment value of +1. The scale variable of this GT has been initially set to 1. By varying the pixel angle in GT, 180 Gabor Transformed Images (MTI) have been obtained. Choosing the maximum magnitude of each MTI produces the final unique Gabor Cervigram Image (GCI).

Fig. (**3**) is the obtained GCI through the various pixel angle-based GT in this article.

### 3.3 Multi Level Features (MLF)

The features correlate the relationship of each pixel in the transformed cervigram image and extract these correlation properties for further pixel classification process. These features are categorized into single-level features and multi-level features. The cervigram detection rate with single-level features is significantly low when compared with the cervigram detection rate with multi level features in this article. This research work uses Local Binary Pattern (LBP) (Rajmohan *et al*. 2022) [36], Local Ternary Pattern (LTP) (Prasad *et al*. 2024), Grey Level Co-occurrence Matrix (GLCM) (Lu *et al*. 2009) and Non-Subsampled Contourlet Transform (NSCT) [37]. These features are called as Texture Pattern Features (TPF) which represents the texture nature of the pixels in the transformed cervigram image. The features are explained in the following sub-section.

The LBP values are obtained for the entire GCI pixels based on the comparison of each center pixel with its surrounding set of pixels. The LBP values are based on the Pattern Window (PW) which has the size of 3 by 3 and this PW has been applied over the entire CGI image. Within PW, the center pixel of PW is compared with its surrounding eight pixel values to produce the unique pattern value. If the surrounding value is having higher value than the center pixel in PW, then ‘0’ is produced, else ‘1’ is produced. After this, all these eight binary values are converted into a decimal value which represents the pattern value of the specific center pixel. Similarly, this procedure has been applied to the entire CGI to obtain the LBP values.

The obtained LBP image from the GCI image has been depicted in Fig. (**4**), where each pixel in LBP image represents the texture feature.

In case of LTP texture feature values, the two feature values have been generated for each pixel in GCI image using trinary condition. Further, GLCM matrix have been constructed at 45 degrees of orientation of pixel values in GCI image and the energy, correlation, entropy, and homogeneity are obtained from this 45-degree oriented GLCM matrix. Moreover, the entire GCI image has been decomposed into two stages using NSCT which has been constructed by low and high pass filter banks. In first stage, higher-order decomposed coefficients are obtained and the lower-order decomposed coefficients are obtained at stage 2. Both lower and higher decomposed set of coefficients have been concatenated into the Contourlet vector.

After obtaining all four features, these features are integrated into a 2D feature matrix whose rows and columns are represented by M by N. This 2D feature matrix has been fed into the proposed CervixNet architecture to obtain the cervigram classification results in this article.

### 3.4 Classification

The computed 2D Matrix Features (FM) are fed into the proposed CervixNet classification architecture for classifying the test cervigram into either a healthy cervigram or a cancer cervigram. This proposed CervixNet architecture has been derived from the existing AlexNet classification architecture by altering the internal modules or layers into parallel. This CervixNet contains two parallel layering modules which are called Internal Layering Level-1 (ILL-1) and Internal Layering Level-2 (ILL-2), as illustrated in Fig. (**5**). Both ILL contains Convolutional layers and Pooling layers with different sets of functional properties.

The ILL-1 contains four consecutive Convolutional Layers (Conv2D) with two pooling layers (Maxpool). In this ILL-1, the Convolutional layers and their pooling layers are illustrated by the following mathematical equations.

**Table:** 

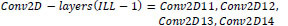	(9)

Where the kernels in each Conv2D of the above equations are given in below equations.

**Table:** 

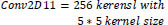	(10)

**Table:** 

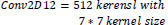	(11)

**Table:** 

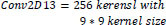	(12)

**Table:** 

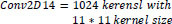	(13)

The main function of this kernel in Conv2D is to perform the linear Convolution process between the computed MF from the features and produces more non-linear features to improve the cervigram detection rate. The number of generated features through the Conv2D decides the cervigram detection rate where this feature count is directionally proportional to the cervigram detection rate. Hence, it is important to have more number of these Conv2D features through the number of kernels. Though the number of Conv2D features improves the cervigram detection rate, it also degrades the functional detection time of the cervical cancer detection system due to its feature matrix size. Therefore, it is necessary to compress the Conv2D features or to reduce the feature matrix size. This is accomplished by the Pooling layers (Maxpool) in the proposed CervixNet.

In this ILL-1, the pooling layers (Maxpool) are illustrated by the following mathematical equations.

**Table:** 

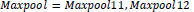	(14)

In general, polling layers have two different pooling functions as which are based on the pooling process. They are Mean Pooling Process (MPP) and Max Pooling Process (MaPP). The MPP performs the feature matrix size reduction by performing the averaging function on all the elements in the Conv2D output. The MaPP performs the feature matrix size reduction by performing the Maximum selection function on all the elements in the Conv2D output. During the retention of the pixels, the MPP process produces the errors which are propagated by the next layers in the CervixNet. There are no significant errors in MaPP process during the pixel retention process. Hence, the MaPP process has been chosen as the best Pooling function and it is adapted to all the Maxpool layers in the proposed CervixNet.

The ILL-2 contains four consecutive Convolutional Layers (Conv2D) with two pooling layers (Maxpool). In this ILL-2, the Convolutional layers and its pooling layers are illustrated by the following mathematical equations.

**Table:** 

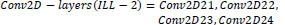	(15)

Where the kernels in each Conv2D of the above equations are given in below equations.

**Table:** 

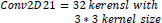	(16)

**Table:** 

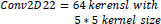	(17)

**Table:** 

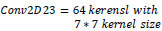	(18)

**Table:** 

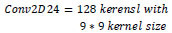	(19)

The Convolutional layers in ILL-1 have being high number of filter kernels which produce certain high matrix features with its large kernel window. The Convolutional layers in ILL-2 have being low number of filter kernels which produces certain low matrix features with its large kernel window. These high and low matrix features are concatenated by the Feature Concatenator (FC) as illustrated in the below equation.

**Table:** 

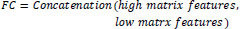	(20)

The size of FC is set by M*N, where M represents the row matrix and N represents the column matrix.

By concatenating these low and high matrix features, the M*N-sized FC size is enlarged by its concatenated feature values. Therefore, the FC has been transferred through the Maxpool23 which reduces the size of the FC more significantly for a further dense layering process. In this proposed CervixNet, three dense layers with unbiased neurons have been used which are illustrated by the following equation.

**Table:** 

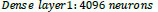	(21)

**Table:** 

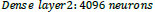	(22)

**Table:** 

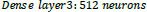	(23)

At the end of this dense layering, all the neurons in Dense layer 3 are merged to produce the final desired output. Based on the value of the produced desired output, the cervigram image has been classified into either a healthy or cancer image as illustrated in the below equation.

**Table:** 

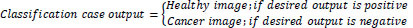	(24)

Fig. (**6a**) belongs to the healthy cervigram image (non-cancer cervigram image) and Fig. (**6b**) belongs to the cancer cervigram image which is obtained from the proposed CervixNet architecture in this article.

The morphological opening image has been obtained from the cancer-classified cervigram image by applying dilation and the morphological closing image has been obtained from the cancer-classified cervigram image by applying erosion. Now, the erosion image has been subtracted from the dilated image to obtain the region of cancer pixels in the final cervigram image.

Fig. (**7a**) is the source cervigram image and Fig. (**7b**) is the segmented cancer region of pixels in the cancer cervigram image.

The pseudo-code of the proposed algorithm is given below.

**Input:** Cervigram image;

**Output:** Cancer region segmented cervigram image;

**Start;**

1. The source cervigram image has been denoised using the proposed DNRF which detects and removes the noise components in the cervigram image;

2. Apply GMT on the noise-reduced cervigram image for the conversion of the spatial into multi-class frequency patterns.

3. Determine the multi-level features from the GMT transformed cervigram image.

4. Form a 2D feature matrix using the determined multi-level features.

5. Feed this 2D feature matrix into the proposed CervixNet classifier with the trained patterns.

6. The classification results have been obtained from the CervixNet classifier using the trained patterns.

7. Apply morphological progress algorithm on the abnormal cervical image to locate the cancer pixels.

## 4 RESULTS AND DISCUSSIONS

This article uses the cervigram images which are available from the open-access databases Guanacaste and Intel and Mobile ODT Cervical Cancer Screening (IMODT). The cervigram images in these databases are obtained from different age groups in different geographic regions around the world. The IMODT database contains 9515 cervigram images which are categorized into type1, type2, type3, and normal. The type1 category is a mild case which contains 1241 cervigram images, type2 category is a moderate case which contains 4348 cervigram images and the type3 category is a severe case which contains 2426 cervigram images and also contains 1500 healthy cervigram images. All these cervigram images have an image size of 1500*1500 pixels as image width and height respectively. Therefore, this database contains 8015 cancer cervigram images and 1500 healthy cervigram images.

The Guanacaste database is originated and maintained by the American National Cancer Institute (ANCI). The cervigram images in this database are obtained from 7000 women patients in South America. From these 7000 women patients, 44000 cervigram images have been obtained and these images have a resolution size of 2891*1973 pixels as image width and height respectively. The cervigram images in this database are categorized into various types as CIN1, CIN2, CIN3, CIN4 and healthy. The CIN1 type belongs to mild category which contains 2300 cervigram images, CIN2 type belongs to a moderate category which contains 400 cervigram images, CIN3 type belongs to a severe category which contains 450 cervigram images and CIN4 type is belonging to a highly severe category which contains 100 cervigram images and also this database contains 490 healthy type cervigram images. Therefore, this database contains 3250 cancer cervigram images and 490 healthy cervigram images.

Along with these cervigram datasets, MATLAB R2021 is used in this paper for obtaining the simulation results and the simulation has been carried out in Intel i7 core processor with 16 GB memory units.

Table **1** is the cervigram image category in IMODT and Guanacaste databases.

The performance of the cervical cancer detection system has been evaluated concerning the number of correctly detected healthy cervigram images and the correctly detected cancer cervigram images. The Healthy Cervigram Detection Index (HCDI) is a computational parameter that is defined as the ratio between the correctly detected healthy cervigram image count and the total healthy cervigram image count. The Cancer Cervigram Detection Index (CCDI) is a computational parameter that is defined as the ratio between the correctly detected cancer cervigram image count and the total cancer cervigram image count. These computational parameters have been evaluated on both cervigram databases and they are depicted in the following equations.

**Table:** 

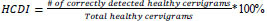	(25)

**Table:** 

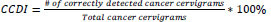	(26)

The proposed cervical cancer detection system obtains 99.3% HCDI by correctly detecting 1490 healthy cervigrams over 1500 healthy cervigrams and also obtains 99.8% CCDI by correctly detecting 8001 cancer cervigrams over 8015 cancer cervigrams for the cervigrams in IMODT database. The average Detection Index (DI) for the cervigrams in this IMODT database is about 99.5%.

The proposed cervical cancer detection system obtains 98.3% HCDI by correctly detecting 482 healthy cervigrams over 490 healthy cervigrams and also obtains 99.7% CCDI by correctly detecting 3241 cancer cervigrams over 3250 cancer cervigrams for the cervigrams in Guanacaste database. The average Detection Index (DI) for the cervigrams in this Guanacaste database is about 99%.

The GMT on the cervigram images creates a greater impact in DI due to its directional sensitivity concerning its spatial-frequency pixel property. This improves the DI value in both database cervigram images. Hence, it is necessary to compare the other transformational models with the GMT on the DI computational process. Table **2** is the impact analysis of transformational models on cervigrams.

The proposed cervical cancer detection system using GMT obtains 99.5% DI in IMODT database and obtains 99% DI in Guanacaste database. The cancer detection system is evaluated with various transformational models instead of GMT in this article to evaluate the computational parameters as illustrated in Table **2**. The Discrete Wavelet Transform (DWT) obtains 95.2% DI in IMODT database and obtains 95.1% DI in Guanacaste database. The Contourlet obtains 94.7%DI in IMODT database and obtains 94.9% DI in Guanacaste database. The Curvelet obtains 94.3%DI in IMODT database and obtains 93.8% DI in Guanacaste database.

In addition with the computational parameters HCDI and CCDI, the following equations are also used in this article to evaluate the computational efficiency of the system.

**Table:** 

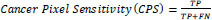	(27)

**Table:** 

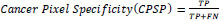	(28)

**Table:** 

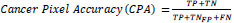	(29)

Whereas, TP and TN belong to the cancer pixels and non-cancer pixels correctly located and FP and FN belong to the cancer pixels and non-cancer pixels incorrectly located.

Table **3** is the computational analysis of cancer pixel detection in IMODT Cervigram images. Here, the ten cervigram images are randomly selected from the abnormal cervigrams in the IMODT database and named from I1 to I10. The proposed methodology has been applied to those randomly chosen ten cervigram images to obtain the unbiased performance estimation. This research work obtains 98.69% CPS, 98.76% CPSP and 99.27% CPA for the set of cervigram images in IMODT database. The same computational values have been obtained for all the cervigrams in this database.

Table **4** is the computational analysis of cancer pixel detection in Guanacaste Cervigram images. Here, the ten cervigram images are randomly selected from the abnormal cervigrams in Guanacaste database and named from G1 to G10. The proposed methodology has been applied on those randomly chosen ten cervigram images to obtain the unbiased performance estimation. This research work obtains **99.22**% CPS, **99.03**% CPSP and **99.01**% CPA for the set of cervigram images in Guanacaste database. The same computational values have been obtained for all the cervigrams in this database.

Table **5** is the comparative analysis of abnormal cervigrams on IMODT database against other existing works Ahishakiye *et al*. (2024), Mathivanan *et al*. (2024), Qathrady *et al*. (2024), Sahay *et al*. (2024), ShtwaiAlsubai *et al*. (2023), Sehra *et al*. (2023), Saini *et al*. (2022), Umesh Kumar Lilhore *et al*. (2022) and Zaid Alyafeai *et al*. (2020). The methodology Ahishakiye *et al*. (2024) obtained 97.18% CPS, 97.10% CPSP, and 97.86% CPA. The methodology Mathivanan *et al*. (2024) obtained 97.09% CPS, 96.98% CPSP and 97.03% CPA. The methodology Qathrady *et al*. (2024) obtained 96.16% CPS, 96.10% CPSP and 96.76% CPA [15]. The methodology Sahay *et al*. (2024) obtained was 96.09% CPS, 95.98% CPSP and 95.67% CPA [17]. ShtwaiAlsubai *et al*. (2023) obtained 95.29% CPS, 96.39% CPSP and 95.30% CPA [26]. The methodology Sehra *et al*. (2023) obtained 94.27% CPS, 95.86% CPSP and 94.57% CPA. The methodology Saini *et al*. (2022) obtained 94.20% CPS, 94.47% CPSP and 93.29%CPA [30]. The methodology Umesh Kumar Lilhore *et al*. (2022) obtained 93.97% CPS, 93.29% CPSP and 93.98%CPA [29]. The methodology Zaid Alyafeai *et al*. (2020) obtained 95.29% CPS, 94.67% CPSP and 94.29%CPA [31].

Table **6** is the comparative analysis of abnormal cervigrams on Guanacaste database against other existing works Ahishakiye *et al*. (2024), Mathivanan *et al*. (2024), Qathrady *et al*. (2024), Sahay *et al*. (2024), Shtwai Alsubai *et al*. (2023), Sehra *et al*. (2023), Saini *et al*. (2022), Umesh Kumar Lilhore *et al*. (2022) and Zaid Alyafeai *et al*. (2020) [13-15, 17, 26, 28-31]. The methodology Ahishakiye *et al*. (2024) obtained 98.10% CPS, 98.45% CPSP, and 98.15% CPA [13]. The methodology Mathivanan *et al*. (2024) obtained 98.14% CPS, 98.54% CPSP and 98.16% CPA [14]. The methodology Qathrady *et al*. (2024) obtained 98.03% CPS, 98.12% CPSP and 98.06% CPA [15]. The methodology Sahay *et al*. (2024) obtained 97.28% CPS, 97.87% CPSP and 97.67% CPA [17]. The methodology Shtwai Alsubai *et al*. (2023) obtained 97.20% CPS, 96.39% CPSP and 96.28% CPA [26]. The methodology Sehra *et al*. (2023) obtained 96.75% CPS, 96.20% CPSP and 95.95% CPA [28]. The methodology Saini *et al*. (2022) obtained 95.30% CPS, 95.95% CPSP and 94.39%CPA [30]. The methodology Umesh Kumar Lilhore *et al*. (2022) obtained 94.28% CPS, 94.57% CPSP and 94.98%CPA [29]. The methodology Zaid Alyafeai *et al*. (2020) obtained 94.29% CPS, 94.08% CPSP and 93.57%CPA [31].

## CONCLUSION

The modified deep learning architecture CervixNet has been proposed in this article to detect the cancer cervical images from the healthy cervical images. The overfitting problem in the conventional deep learning algorithms has been overcome by the proposed CervixNet classification architecture stated in this article, which is the main reason for obtaining the higher cervical image classification results. This method detects and segments both the inter and exterior pixel boundaries of the cancer region which improves the performance efficiency of this method. The proposed cervical cancer detection system obtains 99.3% HCDI by correctly detecting 1490 healthy cervigrams over 1500 healthy cervigrams and also obtains 99.8% CCDI by correctly detecting 8001 cancer cervigrams over 8015 cancer cervigrams for the cervigrams in IMODT database. The average Detection Index (DI) for the cervigrams in this IMODT database is about 99.5%. The proposed cervical cancer detection system obtains 98.3% HCDI by correctly detecting 482 healthy cervigrams over 490 healthy cervigrams and also obtains 99.7% CCDI by correctly detecting 3241 cancer cervigrams over 3250 cancer cervigrams for the cervigrams in Guanacaste database. The average Detection Index (DI) for the cervigrams in this Guanacaste database is about 99%. This research work obtains 98.69% CPS, 98.76% CPSP and 99.27% CPA for the set of cervigram images in IMODT database. This research work obtains 99.22% CPS, 99.03% CPSP, and 99.01% CPA for the set of cervigram images in Guanacaste database. In the future direction of this research work, the severity stages of the segmented cancer regions in the cervigram will be analyzed using Generative Adversarial Networks (GAN).

## Figures and Tables

**Fig. (1):**
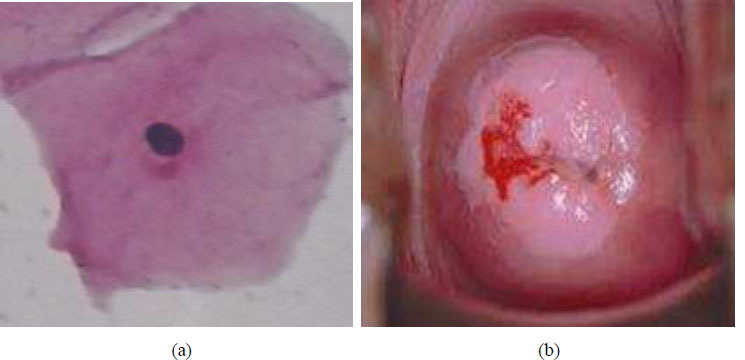
(**a**) Pap smear cell image (**b**) Cervigram.

**Fig. (2):**
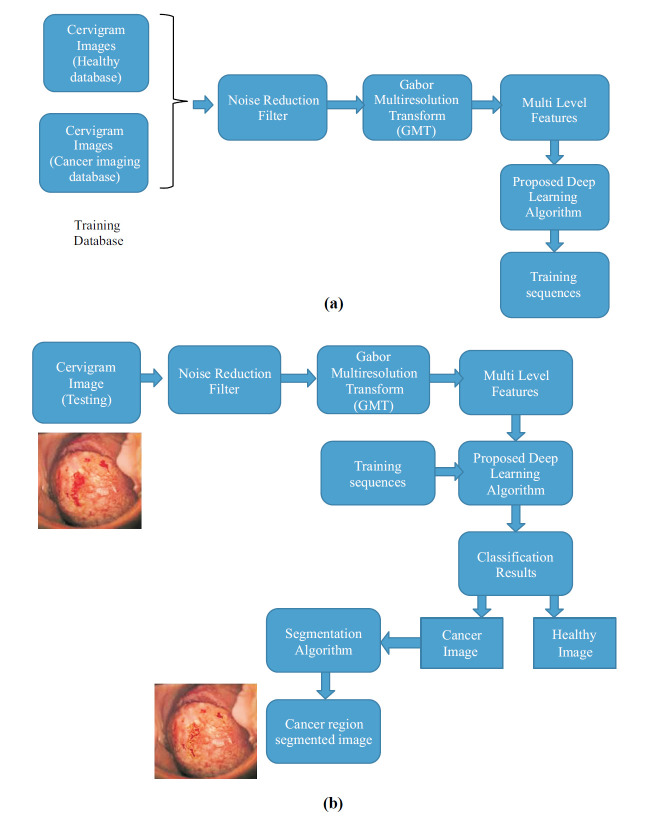
(**a**) Cervigram image classification system in case of training process (**b**) Cervigram image classification system in case of testing process.

**Fig. (3):**
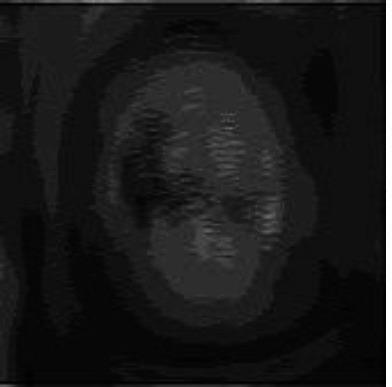
GCI.

**Fig. (4):**
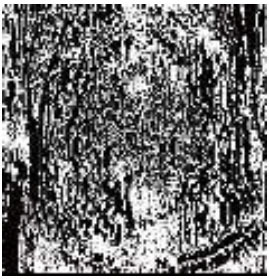
LBP.

**Fig. (5):**
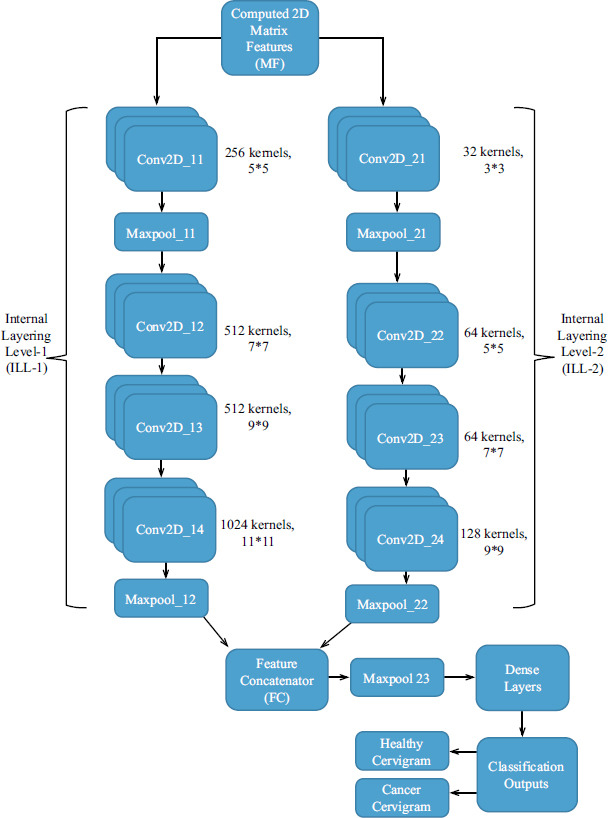
Proposed CervixNet architecture for the cervigram classifications.

**Fig. (6):**
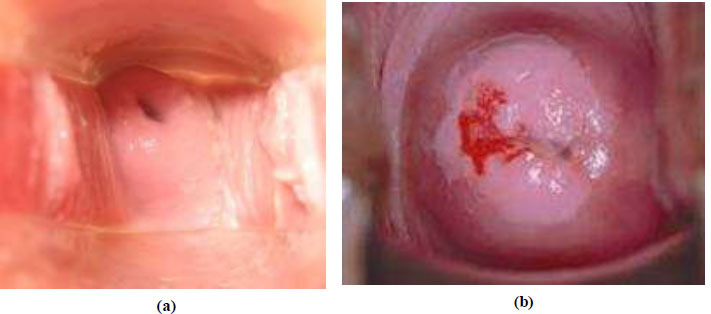
(**a**) Healthy cervigram image (**b**) Cancer cervigram image.

**Fig. (7):**
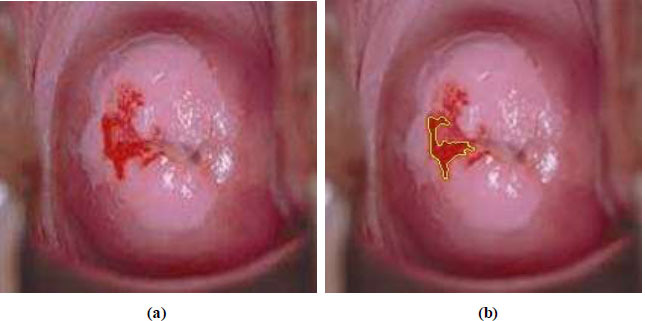
(**a**) Source cervigram (**b**) Segmented cancer region of pixels in cancer cervigram.

**Table 1:** Cervigram image category in IMODT and guanacaste databases.

**Cervigram Database**	**Cervigram Image Category**		**Number of Cervigram Images**
IMODT	Normal	Healthy category	1500
	Type1	Cancer category	1241
	Type2		4348
	Type3		2426
Guanacaste	Normal	Healthy category	490
	CIN1	Cancer category	2300
	CIN2		400
	CIN3		450
	CIN4		100

**Table 2:** Impact analysis of transformational models on cervigrams.

**Transforms**	**DI in %**	
	**IMODT Database**	**Guanacaste Database**
GMT (Proposed)	99.5	99
DWT	95.2	95.1
Contourlet	94.7	94.9
Curvelet	94.3	93.8

**Table 3:** Computational analysis of cancer pixel detection in IMODT cervigram images.

**Cervigrams**	**Numerical Values in %**		
	**CPS**	**CPSP**	**CPA**
I1	98.3	98.4	98.5
I2	98.2	98.8	98.2
I3	99.4	99.3	99.4
I4	98.3	99.1	99.2
I5	98.7	98.8	99.7
I6	98.4	98.5	99.4
I7	98.2	98.3	99.6
I8	98.9	98.7	99.8
I9	99.4	99.3	99.3
I10	99.1	98.4	99.6
Average values	98.69	98.76	99.27

**Table 4:** Computational analysis of cancer pixel detection in guanacaste cervigram images.

**Cervigrams**	**Numerical Values in %**		
	**CPS**	**CPSP**	**CPA**
G1	99.3	99.4	98.7
G 2	99.2	99.3	99.4
G 3	99.6	98.9	99.3
G 4	98.9	98.6	99.6
G 5	99.4	99.4	98.9
G 6	99.3	99.3	99.4
G 7	99.6	98.7	99.2
G 8	99.9	98.9	98.6
G 9	98.6	98.5	98.3
G 10	98.4	99.3	98.7
Average values	99.22	99.03	99.01

**Table 5:** Comparative analysis of abnormal cervigrams on IMODT database against other existing works.

**Methods/Refs**	**Numerical Values in %**		
	**CPS**	**CPSP**	**CPA**
Proposed work	98.69	98.76	99.27
Ahishakiye *et al*. (2024) [13]	97.18	97.10	97.86
Mathivanan *et al*. (2024) [14]	97.09	96.98	97.03
Qathrady *et al*. (2024) [15]	96.16	96.10	96.76
Sahay *et al*. (2024) [17]	96.09	95.98	95.67
Shtwai Alsubai *et al*. (2023) [26]	95.29	96.39	95.30
Sehra *et al*. (2023) [28]	94.27	95.86	94.57
Saini *et al*. (2022) [30]	94.20	94.47	93.29
Umesh Kumar Lilhore *et al*. (2022) [29]	93.97	93.29	93.98
Zaid Alyafeai *et al*. (2020) [31]	95.29	94.67	94.29

**Table 6:** Comparative analysis of abnormal cervigrams on guanacaste database against other existing works.

**Methods/Refs**	**Numerical Values in %**		
	**CPS**	**CPSP**	**CPA**
Proposed work	99.22	99.03	99.01
Ahishakiye *et al*. (2024) [13]	98.10	98.45	98.15
Mathivanan *et al*. (2024) [14]	98.14	98.54	98.16
Qathrady *et al*. (2024) [15]	98.03	98.12	98.06
Sahay *et al*. (2024) [17]	97.28	97.87	97.67
Shtwai Alsubai *et al*. (2023) [26]	97.20	96.39	96.28
Sehra *et al*. (2023) [28]	96.75	96.20	95.95
Saini *et al*. (2022) [30]	95.30	95.95	94.39
Umesh Kumar Lilhore *et al*. (2022) [29]	94.28	94.57	94.98
Zaid Alyafeai *et al*. (2020) [31]	94.29	94.08	93.57

## Data Availability

The data and supportive information are available within the article.

## LIST OF ABBREVIATIONS

HM: Heuristic Metric
DNRF: Directional Noise Reduction Filter
HPV: Human Papilloma Virus
CPS: Cancer Pixel Sensitivity
CPSP: Cancer Pixel Specificity
CPA: Cancer Pixel Accuracy
